# Dental Anxiety Among Children Living in an Orphanage Compared to Children Living with Both of Their Parents in Saudi Arabia: A Case–Control Study

**DOI:** 10.3390/healthcare14121751

**Published:** 2026-06-17

**Authors:** Yazeed Thamer Alshobaili, Rana Abdullah Alamoudi, Mohammed Jamal Barry, Sara Mustafa Bagher, Heba Jafar Sabbagh

**Affiliations:** 1 Ministry of Health, Riyadh 11176, Saudi Arabia; yalshobaili@moh.gov.sa; 2 Pediatric Dentistry Department, Faculty of Dentistry, King Abdulaziz University, Jeddah 21589, Saudi Arabia; rasalamoudi@kau.edu.sa (R.A.A.); mjbarry@moh.gov.sa (M.J.B.); sbagher@kau.edu.sa (S.M.B.); 3 Ministry of Health, Al-Madinah 42351, Saudi Arabia

**Keywords:** adverse family experiences, orphanage, Saudi Arabia, dental anxiety, orphanages, institutionalized

## Abstract

**Background/Objectives**: Dental anxiety (DA) is a well-known obstacle affecting dental care in children. Children living in orphanages are a special population with healthcare needs. The aim of the study was to assess DA among children living in orphanages compared to those living with both biological parents. **Methods**: This frequency-matched case–control study included 61 children living in orphanages in Jeddah city and 122 age- and gender-matched peers living with both parents in Jeddah, Saudi Arabia. Demographic and background data, including medical history, dental visit history, and Adverse Family Experiences (AFEs), were completed by the caregiver. Dental anxiety was assessed subjectively using the self-reported Abeer Children Dental Anxiety Scale (ACDAS) and objectively by the Venham Clinical Anxiety Rating Scale (VCARS). **Results**: The prevalence of children with DA in the study sample among those living in orphanages was 18%. AFEs were significantly higher among children living in orphanages (96.7% vs. 32%, *p* < 0.001). ACDAS and VCARS showed fewer children with DA living in orphanages compared to children living with both parents. Logistic regression showed that living in orphanages decreased the adjusted odds ratio (AOR) of dental anxiety according to ACDAS (AOR = 0.36; *p* = 0.06) and VCARS (AOR = 0.43, *p* = 0.040). **Conclusions**: Although children living in orphanages presented with lower DA than those living with both parents, this may point to differences in emotional expression rather than true emotional state. Clinicians should not rely only on behavioral observations when treating institutionalized children.

## 1. Introduction

Dental anxiety (DA) is an excessive anticipated fear associated with dental visits, often resulting in treatment avoidance and a cycle of oral neglect. Dental fear, on the other hand, is a negative emotional response triggered by specific aspects of the dental visit that leads to an unwanted reaction in the child. Globally, the prevalence of DA has been reported to range from 10 to 30% [1,2,3], while in Saudi Arabia, it has been reported to range from 6.3 to 61.2% [4,5,6,7,8,9].

Dental anxiety has contributing factors such as negative previous dental experience, age, gender, screen time, and parental anxiety [3]. Other related factors such as screen time exposure, sibling order and nicotine exposure were reported [4,10,11,12].

Negative dental experiences strongly reinforce DA, perpetuating avoidance and poor oral health [9,13]. Jeddy et al. (2018) reported that 33.3% of children cited adverse experiences and 79.7% cited pain as primary causes of DA [13].

Younger children show higher anxiety, which decreases with age as coping skills mature [1,2,3]. Among children aged 6–12 years, 25.8% were found to have DA [2], with significantly higher levels in those below eight years old [14]. Gender differences are well established, with females consistently reporting higher DA [2,8]. Parental dental anxiety is another major influence, with moderate parent–child correlations reported in several studies [15,16,17]. Felemban et al. (2019) reported significantly higher fear scores among children of anxious mothers [15] and Gudipaneni et al. (2024) found that parental anxiety increased the likelihood of untreated decay in their children by 2.5 times [18].

In Saudi Arabia, an orphan is defined as a child who has lost his parents, especially the father, who traditionally is the family’s provider [19,20]. Children who cannot be placed in family-based environments are accommodated in institutional facilities that provide comprehensive support and care [21].

Children living in orphanages could face many disadvantages that affect their health and well-being. Al-Jobair et al. (2013) reported chronic medical conditions in 36% of children living in orphanages compared to 14.4% of children living with their parents (*p* < 0.001), along with poorer oral hygiene and higher caries rates [21]. AlSadhan and Al-Jobair [22] further found that 58.9% of institutionalized orphans had digit-sucking habits. Bagher et al. (2023) reported that they were more affected by congenital heart disease (*p* = 0.029) [23]. In addition, studies have linked adverse childhood experiences to many systemic and mental health issues such as diabetes, depression, anxiety, hypertension, mental illness, and smoking [24,25].

This study aimed to assess the prevalence of dental anxiety among children living in orphanages and to compare it to that of children living with both biological parents in Jeddah, Saudi Arabia, using both a self-reported measure, the Abeer Children Dental Anxiety Scale (ACDAS), and an objective measure, the Venham Clinical Anxiety Rating Scale (VCARS).

## 2. Materials and Methods

Ethical Approval was obtained from the Deanship of Ethical Research at King Abdulaziz University (191-12-22) and the Ministry of Human Resources and Social Development (MHRSD). The study was conducted between September 2023 and November 2024.

There were three orphanage facilities that provided care for orphaned children from the cities of Jeddah, Makkah, and Madinah [26,27]. All eligible children aged 6–12 years were recruited from these three facilities in Jeddah, Saudi Arabia. Thus, these facilities represent the orphanage-based child population served within this regional catchment area.

Matched controls were recruited from the Pediatric Dentistry clinics at King Abdulaziz University Dental Hospital (KAUDH) and Jeddah Medical Center (JED-MED) using a 1:2 case-to-control ratio. Inclusion criteria were children aged 6 to 12 years classified by the American Society of Anesthesiologists (ASA) as ASA I (completely healthy) or ASA II (mild, well-controlled medical conditions) [28]. Exclusion criteria were children living with a single parent or relative, not in the study age group, or we were not able to perform an examination or prophy because of a health issue.

The final sample included 61 children living in orphanages and 122 children living with parents. Children living in orphanages formed the case group, while age- and gender-matched children living with both parents served as controls. Frequency matching was applied to minimize potential confounding factors in the anxiety comparisons.

### 2.1. Sample Size

The sample size was calculated based on previous research examining dental anxiety among orphaned children [29]. The calculation utilized a reported dental anxiety prevalence of 36% among orphaned children living in juvenile homes and 60% among non-orphaned children (odds ratio = 0.38). Assuming an allocation ratio of 1:2 (orphan to non-orphan), a statistical power of 80%, and a 5% level of significance, the minimum required sample size was estimated to be participants comprising children in the orphanage group and in the control group. To enhance statistical robustness and minimize potential sampling bias from clinic-based recruitment, we utilized a comprehensive sampling approach for the case group by including all eligible children residing in the orphanage, which resulted in a final total of 61 children in this group.

### 2.2. Assessment Tools

This study utilized two validated instruments to assess dental anxiety: a subjective self-reporting scale and a clinically evaluated objective tool, as detailed in the methodology described by Barry et al. (2025) [30].

#### 2.2.1. Abeer Children Dental Anxiety Scale (ACDAS)

The ACDAS is a validated self-report scale (subjective) consisting of 13 items assessing children’s emotional responses to common dental scenarios, and developed by Al-Namankany et al. (2012) [31]. Each item is scored on a three-point visual scale: 1 = happy face, 2 = neutral face, and 3 = scared face. In this study, children were shown the three-point visual scale and were then asked to choose one of them based on how they felt regarding the dental scenarios. The total score was obtained by summing all 13 items, yielding a possible range of 13 to 39. A score of 26 or above was classified as “anxious,” while a score below 26 was classified as “non-anxious” [31].

#### 2.2.2. Venham Clinical Anxiety Rating Scale (VCARS)

The VCARS is a validated observational tool (objective) developed by Venham et al. (1977) to assess children’s situational anxiety in dental settings [32]. It consists of six behavior-based levels ranging from zero to five, with higher scores indicating greater anxiety. In this study, the investigator assessed each child’s behavior during examination, and the scores were recoded into a binary outcome: 0 = “not anxious” and 1 to 5 = “anxious” [32,33]. This approach was used to reduce the impact of sparse cell counts on the stability of the analysis, facilitate clinical interpretation, and enable the use of logistic regression analysis. However, it is acknowledged that binary classification may reduce the degree of detail of anxiety assessment.

#### 2.2.3. Adverse Family Experiences (AFEs)

Adverse Family Experience (AFE) was established in 2012 using data to assess a child’s adverse childhood experiences through a family-focused questionnaire [34,35]. The AFE was assessed using a 10-item checklist completed by the caregiver. Each item was coded as one for “yes” and zero for “no” or “don’t know,” with a total score ranging from 0 to 10 for each child [34,35]. For children living in orphanages, parental absence or loss was considered a recognized AFE category; therefore, all orphanage participants were classified as having experienced at least one AFE. Non-disclosure of the specific type of adversity by educators, whether due to privacy concerns or uncertainty, was not interpreted as absence of AFE exposure.

AFE assessment in the orphanage group was used to describe available background information rather than to determine whether adversity was present. In contrast, AFEs were assessed among children living with their parents to identify whether similar psychosocial exposures were present in the comparison group. This allowed a clearer description of the exposure profile across groups. Dental anxiety was considered a situational anxiety response related to the dental setting, whereas AFEs were considered background psychosocial exposures that may influence emotional regulation, stress reactivity, and vulnerability to anxiety-related responses.

### 2.3. Pilot Testing and Reliability

A pilot study including 30 children was conducted before data collection to assess the clarity, comprehension, and applicability of the questionnaire. Based on the pilot findings, minor modifications were made to improve the clarity and applicability of the complete data collection form. Following the pilot study and final modification of the data collection form, content validity was assessed. The content validity index demonstrated excellent agreement, with a CVI of 100%. Test–retest reliability was also conducted to assess the consistency of the study instruments [36]. The questionnaire was administered twice, with a one-week interval, to a sample of 10 caregivers attending the pediatric dentistry clinics at King Abdulaziz University Dental Hospital (KAUDH). The intraclass correlation coefficient demonstrated acceptable reliability (ICC = 0.75). In addition, examiner reliability was assessed by an expert in the field; inter-rater reliability was assessed for the Venham clinical examination, and the kappa score demonstrated strong agreement (κ = 0.85).

### 2.4. Data Collection Procedures

After approval of the research was obtained from the hospital and the orphanage administration, scheduling for and transportation to the dental hospital for all orphans were conducted. Informed consent was obtained from the children’s caregivers. For children living in orphanages, consent was obtained from the authorized institutional representatives acting as legal guardians in accordance with regulations of the Ministry of Human Resources and Social Development. Demographic and background data, including age, gender, medical history, dental visit history, and AFEs, were recorded for both groups before the dental examination. The questionnaire was completed by the child’s caregiver, who was either an educator for children living in orphanages or a parent for children living with both their parents, through a structured interview by a trained pediatric dentist. In addition, the ACDAS was administered directly to the child in the waiting room prior to the dental examination. During the dental examination, the VCARS was scored by a single trained and calibrated attending clinician. A single trained pediatric resident provided the examination for all the participating children. Children were interviewed individually without caregivers to minimize social desirability and stigma.

### 2.5. Statistical Analysis

The statistical analysis was performed using the Statistical Package for the Social Sciences (SPSS), version 30 (IBM Corp., Armonk, NY, USA), for Mac OS X [36]. Descriptive statistics, including frequencies and percentages, were calculated for qualitative variables. Categorical variables were compared using the Chi-square test or Fisher’s exact test when expected cell counts were less than 5. Binary logistic regression analyses were conducted to assess dental anxiety as the dependent variable, measured using ACDAS and VCARS. Child living status, categorized as living in an orphanage versus living with parents, was included as the main predictor variable. Age and gender were included as potential confounders. In addition, a generalized linear mixed model was used to examine the association between ACDAS dental anxiety and the study predictors while accounting for the matched case–control design. The threshold for statistical significance was set at *p* ≤ 0.05.

## 3. Results

A total of 68 children were recruited from the three orphanages. However, seven children were excluded for the following reasons: three had an ASA III classification, two were older than the required age range (>12 years), and two were younger than the required age range (<7 years). After exclusions, 61 children living in orphanages met the inclusion criteria, of whom 11 children had dental anxiety, reflecting a prevalence of 18%.

These participants were then matched with children living with their parents based on age, gender, and time of visit. A total of 122 children living with their parents were included. Therefore, the final study sample consisted of 183 children, of whom 61 (33.3%) were living in orphanages and 122 (66.7%) were living with both their parents. Overall, 123 (67.2%) were male, and 60 (32.8%) were female. Of the total sample, 26.2% were ages 6–7 years, 14.8% ages 8–9 years, and 59% ages 10–12 years. AFE was statistically significantly higher among children living in orphanages (96.7%) than children with both their parents (32%) (*p* < 0.001). Regarding previous dental visits, 45 (73.8%) children living in orphanages and 120 (98.4%) children with parents reported a previous dental visit. This difference was statistically significant (*p* < 0.001). See Table 1.

The prevalence of dental anxiety among 7-to-12-year-old children living in orphanages was 18% (11 children). Table 2 shows the distribution of the study groups according to their dental anxiety (see Appendix A, Table A1 for the detailed VCARS score distribution). There were fewer children living in orphanages who were classified as anxious (18.0% according to ACDAS and VCARS, respectively) than children living with both parents (25.4% according to ACDAS and 30.3% according to VCARS), with no statistically significant difference (*p* = 0.263, *p* = 0.075, respectively).

Logistic regression analysis was conducted to assess the relationship between DA, measured as the dependent variable using both the ACDAS and VCARS, and the independent variables of children’s living status, age, and gender (Table 3). Living in an orphanage was associated with a lower AOR of experiencing DA compared to living with both biological parents. This inverse relationship was statistically significant when DA was evaluated using the VCARS (AOR = 0.42; 95% CI: 0.18–0.96; *p* = 0.04), but approached, though did not reach, statistical significance when using the ACDAS (AOR = 0.36; 95% CI: 0.12–1.08; *p* = 0.06).

Regarding age, children aged 6–7 years were significantly more likely to experience DA than older children aged 10–12 years across both assessment scales (ACDAS: AOR = 3.79; 95% CI: 1.54–9.30; *p* = 0.007; VCARS: AOR = 10.42; 95% CI: 4.27–25.30; *p* < 0.001). Furthermore, gender was significantly associated with DA; males had significantly lower odds of experiencing DA compared to females based on the ACDAS (AOR = 0.13; 95% CI: 0.05–0.31; *p* < 0.001). A similar trend was observed using the VCARS, though it did not reach statistical significance (AOR = 0.49; 95% CI: 0.22–1.08; *p* = 0.2).

In the mixed-effects logistic regression model accounting for age- and gender-matched clusters, orphans had lower AOR of no-anxiety (ACDAS = 0.425 and VCARS = 0.134) compared to children living with their parents. This relationship was not statistically significant for VCARS (*p* = 0.496 and 95% CI [0.12, 1.46]) and ACDAS (*p* = 0.174 and 95% CI [0.12, 1.46]).

## 4. Discussion

This is a prevalence and case–control study that explored dental anxiety among children living in orphanages compared with age- and gender frequency-matched children living with both parents in Saudi Arabia. By using both a subjective self-reported tool (ACDAS) and an objective observational tool (VCARS), children living in orphanages had dental anxiety levels comparable to or lower than those of children living with both parents, despite significantly greater exposure to AFEs reported by caregivers.

This study is considered distinctive in that it assessed dental anxiety among children living in orphanages using both subjective and objective methods. Although Chikkala et al. (2015) [29] conducted a similar study, their work was cross-sectional and was performed within the institutions without an actual dental visit. In addition, their assessment relied solely on a questionnaire and did not include dental exposure or an objective clinical measure. By contrast, in the present study, all children attended a scheduled dental appointment, received prophylaxis before the assessment of dental anxiety, and were included within a frequency-matched case–control design [29]. In addition, this study is the first to examine DA among children living in orphanages in Jeddah, Saudi Arabia.

Moreover, the prevalence of dental anxiety observed in the present study falls within the range reported globally (approximately 10–30%) [1,2,3] and locally in Saudi Arabia by previous studies (6.3–61.2%) [4,5,6,7,8,9]. However, when comparing children living in orphanages with those living with both parents, dental anxiety was higher among the latter. This may be partly related to the fact that the comparison group consisted of hospital-based controls, as previous dental experiences have been associated with dental anxiety in children [37]. Such experiences may either increase or decrease dental anxiety depending on the type of dental procedure received and the behavior management techniques used. Since not all children living in orphanages had previous dental visits, they may have had fewer prior negative dental experiences, which could be associated with a lower likelihood of developing dental fear and anxiety [38,39]. However, in the present study, we attempted to minimize this effect by assessing dental anxiety after procedures common to both groups, namely clinical examination, prophylaxis, and fluoride application. In addition, this finding is consistent with Chikkala et al. (2015), who reported significantly lower dental anxiety among children living in government-run orphanages than among children living with both parents [29].

One explanation for this decrease in dental anxiety is that the absence of parents in the dental setting may reduce the intergenerational transmission of anxiety. Studies have shown moderate to strong correlations between parental fear and anxiety and children’s dental anxiety, mediated through verbal transmission of fear, modeling, and reinforcement of avoidance behavior [15,18]. Therefore, children living with both their parents may paradoxically be more vulnerable to inherited or reinforced DA, particularly when parents themselves harbor unresolved DA and fear.

Another possible explanation for this is that children living in institutional care may experience greater exposure to structured healthcare encounters, including routine medical and dental visits, often accompanied by trained caregivers rather than emotionally anxious parents. Repeated exposure to clinical environments under predictable and supervised conditions may facilitate habituation and desensitization, thereby potentially reducing anticipatory AD. This interpretation is consistent with learning theory models, which propose that anxiety diminishes when repeated exposure occurs in the absence of reinforcement of fear responses [8]. However, these factors were not reported in relation to the study outcomes and therefore cannot be viewed as contributing to the observed findings.

Attachment theory has been proposed as a framework for understanding emotional responses in children exposed to early institutionalization or neglect [40,41]. Children who experience inconsistent caregiving often learn that the expression of distress does not reliably elicit a comforting response. Although this mechanism was not assessed in the present study, it may offer a theoretical framework for interpreting the observed pattern [42]. In the context of a stressful dental environment, the lower anxiety scores observed in the orphanage cohort may not necessarily reflect a true absence of fear but could hypothetically reflect differences in emotional expression. While children living with their parents may express dental anxiety more openly in the expectation of parental support, children living in institutional care may respond differently, potentially resulting in lower scores on standardized behavioral and self-report measures [43]. This may be reflected in the present study, where dental anxiety reached statistical significance in the regression analysis in the dentist-rated clinical measure (VCARS), but not in the self-reported measure (ACDAS). In addition, these psychological processes were not directly assessed in the present study and therefore should be interpreted as hypothetical rather than explanatory mechanisms. Thus, the present study may serve as an initial step in assessing dental anxiety among children exposed to adverse childhood experiences.

Moreover, the findings of this study may be considered in the context of the Saudi Arabian national strategic framework and Vision 2030. This framework outlines a systemic transition from congregate institutional care toward a model of de-institutionalization. This policy prioritizes family-based alternative care as the primary intervention for orphaned children. The overarching objective is to mitigate the documented developmental and psychological risks associated with long-term institutionalization by providing stable, nurturing environments that foster healthier attachment patterns and long-term resilience [27,44,45,46].

In addition, despite the significantly higher proportion of AFEs among children living in orphanages, DA was lower among them compared to children living with both parents. This observation may suggest a distinction between general psychosocial adversity and situation-specific anxiety, such as dental anxiety. While adverse childhood experiences are strongly associated with long-term mental health outcomes, including anxiety and depression, their relationship with DA remains unclear and may be influenced by contextual factors [44,47,48].

Age emerged as a strong predictor of DA, with younger children exhibiting significantly higher odds of DA. This finding is consistent with extensive literature demonstrating that DA decreases with age as children’s cognitive maturity, emotional regulation, and coping strategies improve [2,4,7,14]. Younger children often have a limited ability to contextualize dental procedures, heightened sensory sensitivity, and a greater reliance on emotional reassurance, all of which may amplify responses in unfamiliar clinical environments. As children grow older, increased dental exposure and improved cognitive appraisal of procedures contribute to reduced anxiety [2,4,7,14].

Gender differences were also evident, with female children showing higher DA levels than male children. This pattern mirrors findings across diverse cultural settings and age groups. Epidemiological studies consistently report higher DA prevalence among females, which could potentially reflect biological sensitivity to stress, socialization patterns that allow greater expression of fear, or differences in emotional processing [1,2,5,7,8].

A key strength of this study is that it included all eligible children living in the three orphanage facilities in Jeddah, which provide care for orphaned children from Jeddah, Makkah, and Madinah. Although the number of children living in orphanages was relatively small, this reflects the limited number of children residing in orphanage facilities within this regional catchment area rather than selective recruitment. Therefore, the comprehensive inclusion of eligible orphanage children reduces selection bias within this group and provides a meaningful representation of dental anxiety among children living in orphanage care in this region. Another strength of this study is the use of two dental anxiety assessment tools, ACDAS and VCARS, which allowed dental anxiety to be evaluated from different aspects of the child’s anxiety response, rather than relying on a single instrument. This is especially important in children living in orphanages, as some children may have difficulty expressing fear verbally. Furthermore, although orphans showed an AOR of dental anxiety of 1.59 in the mixed model for ACDAS and 2.14 for Venham, the result was not statistically significant for ACDAS. This suggests a possible association that requires a larger sample size to confirm.

The limitations of the study include the use of a self-reported questionnaire completed by participating children and caregivers, which may introduce recall and reporting bias. In addition, the use of hospital-based controls may have introduced selection bias, as the control group was recruited from a hospital-based pediatric dental clinic. However, this approach was necessary to assess dental anxiety in the same dental setting for both cases and controls. Furthermore, most children in the orphan group had previously received dental treatment, and both groups underwent prophylaxis treatment before the assessment of dental anxiety, which may have reduced the impact of selection bias related to the use of hospital-based controls and decreased the likelihood that participants were entirely unfamiliar with dental care. In addition, adverse family experiences were reported by different informants across groups (parents and institutional caregivers), which may have affected the accuracy and comparability of this information.

For the non-significant finding related to dental anxiety, particularly the ACDAS result that approached statistical significance, a post hoc power analysis was conducted to clarify whether the study had adequate statistical power to detect the observed association. The analysis was performed using G*Power version 3.1.9.7 under the z-test family for logistic regression, applying the large-sample z-test approach with Demidenko’s variance correction. The analysis was based on a two-tailed test, an observed odds ratio of 0.42, a baseline outcome probability of 0.36, an alpha level of 0.05, a total sample size of 183 participants, an $R^{2}$ value of 0.30 for other predictors, and a binomial predictor distribution with π=0.375. The calculated critical z-value was −1.960, and the achieved statistical power was 0.515, indicating that the study had approximately 51.5% power to detect the observed association under the specified model assumptions.

This relatively low power suggests that the non-significant result should be interpreted cautiously. Although the association did not reach statistical significance, the finding may partly reflect limited statistical power rather than definitive evidence of no association. However, because we also used a mixed-effects logistic regression model accounting for age- and gender-matched clusters, the G*Power result should be interpreted as an approximate post hoc power estimate rather than an exact power calculation for the mixed-effects model.

Future research should include multi-center studies across different cities in Saudi Arabia to improve generalizability, as well as longitudinal studies to examine DA, behavior, and emotional problems over time. Finally, investigating how parental and caregiver styles influence children’s anxiety is recommended.

## 5. Conclusions

The prevalence of DA among children living in orphanages was 18%. Although dental anxiety was lower among children living in orphanages compared to those living with both parents after adjusting the odds ratio, this should be interpreted with caution and may reflect differences in emotional expression rather than true emotional state. Clinicians should not rely only on behavioral observations when treating institutionalized children.

## Figures and Tables

**Table 1:** Distribution of the participating children according to age, gender, dental history and history of exposure to an adverse family experience (N = 183).

Variables		Child’s Living Status		Total	*p* Value
		Living in Orphanage *n* (%)	Living with Both Their Parents *n* (%)	*n* = 183 (%)	
Age (years)	6–7	16 (26.2)	32 (26.2)	48 (26.2)	1.0 ^X2^
	8–9	9 (14.8)	18 (14.8)	27 (14.8)	
	10–12	36 (59.0)	72 (59.0)	108 (59.0)	
Gender	Male	41 (67.2)	82 (67.2)	123 (67.2)	1.0 ^X2^
	Female	20 (32.8)	40 (32.8)	60 (32.8)	
Previous dental visit ^F^	Yes	45 (73.8)	120 (98.4)	167 (91.3)	<0.001 *
	No	16 (26.2)	2 (1.6)	16 (8.7)	
History of AFE ^F^	Yes	61 (100)	39 (32)	100 (54.6)	<0.001 *
	No	0	83 (68)	83 (45.4)	

AFE: Adverse family experiences. ^F^: Fisher’s exact test. ^X2^: Chi-square test. * Statistical significance at *p* ≤0.05.

**Table 2:** Distribution of the participating children according to the anxiety status based on the Abeer Children’s Dental Anxiety Scale (ACDAS) and Venham Clinical Anxiety Rating Scale (VCARS) and living status (N = 183).

Dental Anxiety	Response	Child’s Living Status		*p*-Value
		Living in Orphanage *n* (%)	Living with Both Their Parents *n* (%)	
ACDAS	Yes ** No ***	11 (18) 50 (82)	31 (25.4) 91 (74.6)	0.263 ^X2^
VCARS	Yes ** No ***	11 (18) 50 (82)	37 (30.3) 85 (69.7)	0.075 ^X2^
Total		61 (100)	122 (100)	

ACDAS: Abeer Children’s Dental Anxiety Scale. VCARS: Venham Clinical Anxiety Rating Scale score. ^X2^: Chi-square test. ** Anxious when the Abeer Children’s Dental Anxiety Scale ≥ 26 or when the Venham Clinical Anxiety Rating Scale score ranged from 1 to 5. *** Non-anxious when the Abeer Children’s Dental Anxiety Scale < 26 or when the Venham Clinical Anxiety Rating Scale scored zero.

**Table 3:** Regression analysis of the relationship between the child’s anxiety status based on the Abeer Children’s Dental Anxiety Scale/Venham’s Clinical Dental Anxiety Scale (dependent variables) and living status, age, and gender (independent variables) (N = 183).

Variables	ACDAS			VCARS		
	Yes *n* (%)	No *n* (%)	AOR (95% CI) *p*-Value	Yes *n* (%)	No *n* (%)	AOR (95% CI) *p*-Value
Child’s living status						
Living in orphanage	11 (26.2)	50 (35.5)	0.36 (0.12–1.08) 0.06	11 (22.9)	50 (37)	0.42 (0.18–0.96) 0.04 *
Living with both their parents ^R^	31 (73.8)	91 (64.5)	1	37 (77.1)	85 (63)	1
Age group in years						
6–7	18 (42.9)	30 (21.3)	3.79 (1.54–9.30) 0.007 *	27 (56.3)	21 (15.6)	10.42 (4.27–25.3) <0.001 *
8–9	7 (16.7)	20 (14.2)	1.61 (0.74–7.53) 0.38	8 (16.7)	19 (14.1)	3.70 (1.25–10.9) 0.05 *
10–12 ^R^	17 (40.5)	91 (64.5)	1	13 (27.1)	95 (70.4)	1
Gender						
Male	11 (26.2)	112 (79.4)	0.13 (0.05–0.31) <0.001	26 (54.2)	97 (71.9)	0.49 (0.22–1.08) 0.2
Female ^R^	31 (73.8)	29 (20.6)	1	22 (45.8)	38 (28.1)	1
Total	48 (100)	135 (100)		48 (100)	135 (100)	

ACDAS: Abeer Children’s Dental Anxiety Scale. VCARS: Venham Clinical Anxiety Rating Scale score. ^R^: Reference. * Statistical significance at *p* ≤0.05.

## Data Availability

The raw data supporting the conclusions of this article will be made available by the authors on request.

## Abbreviations

The following abbreviations are used in this manuscript:

DA	Dental anxiety
ACDAS	Abeer Children Dental Anxiety Scale
VCARS	Venham Clinical Anxiety Rating Scale
MHRSD	Ministry of Human Resources and Social Development
KAUDH	King Abdulaziz University Dental Hospital
JED-MED	Jeddah Medical Center
ASA	American Society of Anesthesiologists
AFEs	Adverse Family Experiences

## Appendix A

**Table A1:** Distribution of Dental Anxiety and VCARS Scores According to Children’s Living Status.

Score		Children Living Status		*p*-Value
		In Orphanages N (%)	With Parents N (%)	
Child’s Dental Anxiety	Yes ** No ***	11 (18.0) 50 (82.0)	37 (30.3) 85 (69.7)	0.750 ^X2^
Score (0)		50 (82.0)	85 (69.7)	0.030 * ^X2^
Score (1)		8 (13.1)	30 (24.6)	
Score (2)		1 (1.6)	7 (5.7)	
Score (3)		0 (0.0)	0 (0.0)	
Score (4)		2 (3.3)	0 (0.0)	
Score (5)		0 (0.0)	0 (0.0)	
Total		61 (100)	122 (100)	183 (100)

^X2^: Chi-square test. * Statistical significance at *p* ≤0.05. ** Anxious when VCARS score = 1–5. *** when VCARS anxiety score is 0.
